# Apoptosis of tumor infiltrating effector TIM-3+CD8+ T cells in colon cancer

**DOI:** 10.1038/srep15659

**Published:** 2015-10-23

**Authors:** Chiao-Wen Kang, Avijit Dutta, Li-Yuan Chang, Jayashri Mahalingam, Yung-Chang Lin, Jy-Ming Chiang, Chen-Yu Hsu, Ching-Tai Huang, Wan-Ting Su, Yu-Yi Chu, Chun-Yen Lin

**Affiliations:** 1 Graduate Institute of Biomedical Sciences, College of Medicine, Chang Gung University, Kweishan- 333, Taoyuan, Taiwan; 2 Division of Hepatogastroenterology, Department of Internal Medicine, Chang Gung Memorial Hospital and College of Medicine, Chang Gung University, Kweishan- 333, Taoyuan, Taiwan; 3 Division of Infectious Diseases, Department of Medicine, Chang Gung Memorial Hospital and Chang Gung University, Kweishan- 333, Taoyuan, Taiwan; 4 Division of Hematooncology, Department of Medicine, Chang Gung Memorial Hospital and Chang Gung University, Kweishan, 333, Taoyuan, Taiwan; 5 Colorectal Surgery Section, Department of Surgery, Chang Gung Memorial Hospital and Chang Gung University, Kweishan- 333, Tayouan, Taiwan

## Abstract

TIM-3 functions to enforce CD8+ T cell exhaustion, a dysfunctional state associated with the tolerization of tumor microenvironment. Here we report apoptosis of IFN-γ competent TIM-3+ population of tumor-infiltrating CD8+ T cells in colon cancer. In humans suffering from colorectal cancer, TIM-3+ population is higher in cancer tissue-resident relative to peripheral blood CD8+ T cells. Both the TIM-3+ and TIM-3- cancer tissue-resident CD8+ T cells secrete IFN-γ of comparable levels, although apoptotic cells are more in TIM-3+ compared to TIM-3- population. In mouse CT26 colon tumor model, majority of tumor-infiltrating CD8+ T cells express TIM-3 and execute cytolysis function with higher effector cytokine secretion and apoptosis in TIM-3+ compared to TIM-3- population. The tumor cells secrete galectin-9, which increases apoptosis of tumor-infiltrating CD8+ T cells. Galectin-9/TIM-3 signaling blockade with anti-TIM-3 antibody reduces the apoptosis and in addition, inhibits tumor growth in mice. The blockade increases therapeutic efficacy of cyclophosphamide to treat tumor in mice as well. These results reveal a previously unexplored role of TIM-3 on tumor-infiltrating CD8+ T cells *in vivo*.

Cells and molecules of immune system are the fundamental components of anti-tumor immune responses in the tumor microenvironment. CD8+ T cells migrate to tumor sites to eliminate the tumor cells. An increase in the number of tumor-infiltrating CD8+ T cells in the tumor microenvironment correlates with better clinical outcomes of human cancers^1^,^2^,^3^. However, induction of inhibitory signaling pathways (immune checkpoints) during CD8+ T cell response to tumor cells results in CD8+ T cell exhaustion^4^,^5^ that helps in progression of the tumor^6^. Tumor-infiltrating CD8+ T cells express co-inhibitory molecules such as TIM-3 (T cell immunoglobulin and mucin protein 3), which is often co-expresses with PD-1. Both the TIM-3 and PD-1 are now heavily regarded as markers of exhaustion on CD8+ T cells in tumors and in chronic viral infections^7^,^8^,^9^,^10^,^11^,^12^, although TIM-3 was initially identified on functionally active Th1 cells and effector CD8+ T cells^13^,^14^. Ngiow *et al*. has shown that TIM-3 blockade rejuvenates IFN-γ production in several experimental and carcinogen-induced tumors including the mouse CT-26 tumor model^15^. Still the putative reasons for the higher ratio of tumor-infiltrating CD8+: CD4+ T cells with therapy of anti-mouse TIM-3 monoclonal antibody remain elusive^15^. As TIM-3 has affinity to galectin-9 (ref. 16) that induces apoptosis in both human and murine T cells^17^, negative regulation of T cell immunity through TIM-3 signaling^14^,^16^,^17^,^18^ may increase apoptosis of tumor-infiltrating T cells in tumor microenvironment.

Colorectal cancer (CRC) is the third predominant cancer type and a major cause of cancer-related human death worldwide^19^. Immune dysfunction with T cell exhaustion has been postulated as a major reason where cancer tissue-resident CD4+ and CD8+ T cells express TIM-3 and PD-1 on their surface^20^,^21^. As an increase in the number of tumor-infiltrating CD8+ T cells improves clinical outcome of human colorectal cancer^1^,^22^,^23^,^24^, it is possible that tumor-infiltrating T cell deletion with apoptosis imposes immune dysfunction. Through the study of cancer tissue and peripheral blood of colorectal cancer patients, here we reveal a higher share of TIM-3+ cells in the cancer tissue-resident relative to the peripheral blood CD8+ T cells in the same patient. TIM-3 expression and functional exhaustion are not interconnected, as both the TIM-3+ and TIM-3- cancer tissue-resident CD8+ T cells demonstrate comparable IFN-γ response. Moreover, apoptotic cells are of higher proportion in the cancer tissue-resident compared to the peripheral blood CD8+ T cells. In cancer tissue resident CD8+ T cells, apoptotic cells are more in TIM-3+ compared to TIM-3- population. Similar to the humans, majority of the tumor-infiltrating CD8+ T cells express TIM-3 in CT26 mouse colon tumor model. Tumor-bearing mice derived CD8+ T cells execute cytolysis effect on tumor cells *in vitro* and inhibit tumor growth upon adoptive transfer into another tumor-bearing mice. Compared to the TIM-3- population, TIM-3+ CD8+ T cells secrete more effector cytokines such as IIFN-γ, TNF-α and IL-2. Apoptotic cells are higher in tumor-infiltrating relative to splenic CD8+ T cells with TIM-3+ cells in majority. Tumor cells secrete galectin-9, which increases apoptosis of tumor-infiltrating CD8+ T cells. Blockade of TIM-3 by anti- TIM-3 antibody reduces galectin-9 induced apoptosis. The blockade also increases the therapeutic efficacy of cyclophosphamide to treat tumor in mice. Taken together, our results suggest TIM-3 expression do not imply functional exhaustion of tumor-infiltrating CD8+ T cells. Interaction between tumor derived galectin-9 and TIM-3 on the infiltrating CD8+ T cells induce apoptosis in functionally active tumor-infiltrating TIM-3+CD8+ T cells.

## Results

### Apoptosis of IFN-γ competent TIM-3+ cancer tissue resident CD8+ T cells in human colorectal cancer

**Figure 1:**
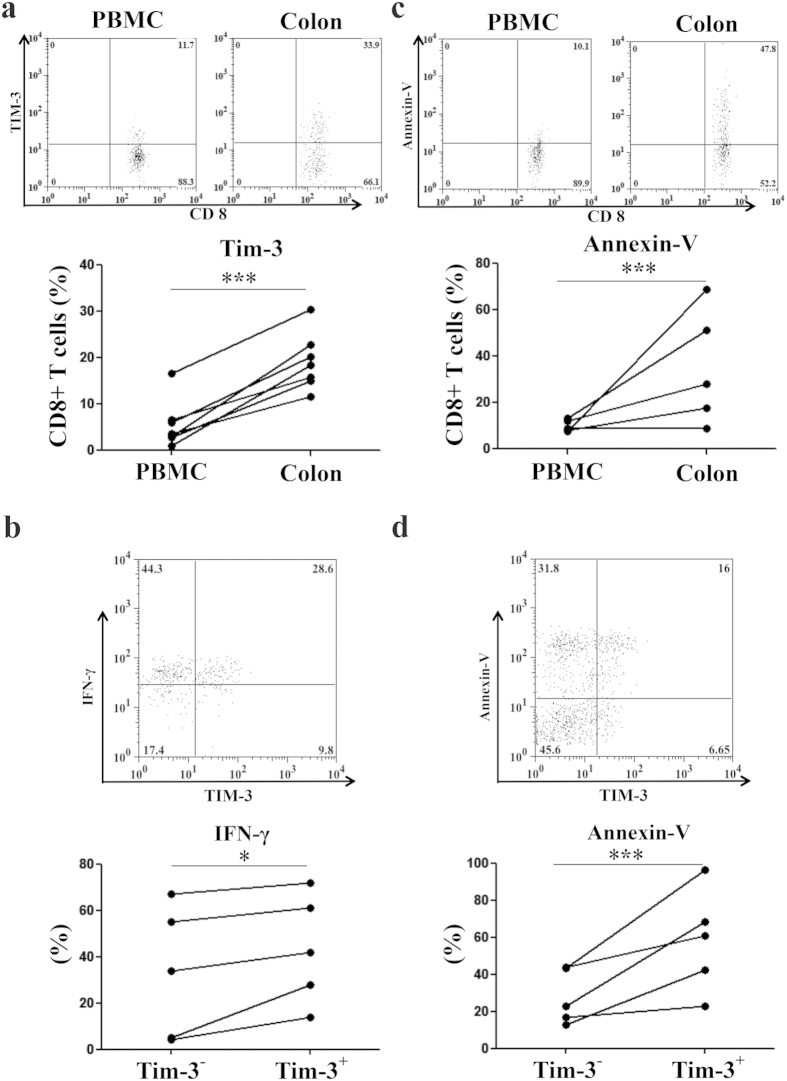
Effector response and apoptosis of cancer tissue resident TIM-3+CD8+ T cells in human colorectal cancer. Cancer tissues and peripheral bloods of the humans suffering from colorectal cancer were analyzed. (**a**) Higher TIM-3 expression by cancer tissue resident relative to peripheral blood CD8+ T cells in same patient. (**b**) Higher IFN-γ response by TIM-3+ compared to TIM-3- cancer tissue resident CD8+ T cells. (**c**) Higher apoptosis of CD8+ T cells in cancer tissue relative to peripheral blood in same patient. (**d**) Higher apoptosis of TIM-3+ compared to TIM-3- cancer tissue resident CD8+ T cells. Dot-plots are representative primary data and each symbol represents individual patient in the graphs. Assessments were confirmed statistically using a paired t-test (*** = p < 0.0001; * = p < 0.01).

We analyzed TIM-3 expression on CD8+ T cells both in the cancer tissues and peripheral bloods in humans suffering from colorectal cancer (CRC) by flow cytometry. The share of TIM-3 expressing cells in CD8+ T cell population was higher in cancer tissue compared to that in peripheral blood of the same CRC patient (Fig. 1a). Among cancer tissue resident CD8+ T cells, TIM-3+ population was equally or more potent for IFN-γ response compared to that by the TIM-3- population (Fig. 1b). Apoptosis of the CD8+ T cells was higher in the cancer tissue relative to the peripheral blood (Fig. 1c) and more importantly, TIM-3 expressing cells were more apoptotic than the TIM-3 non-expressing counterparts in the cancer tissue resident CD8+ T cells of the same CRC patient (Fig. 1d). These results suggest accumulating TIM-3+CD8+ T cells are functionally efficient but prone to death in the cancer tissues of CRC patients.

### TIM-3 and PD-1 expression with T-bet and Eomes co-induction of tumor infiltrating CD8+ T cells in mouse CT26 colon tumor model

**Figure 2:**
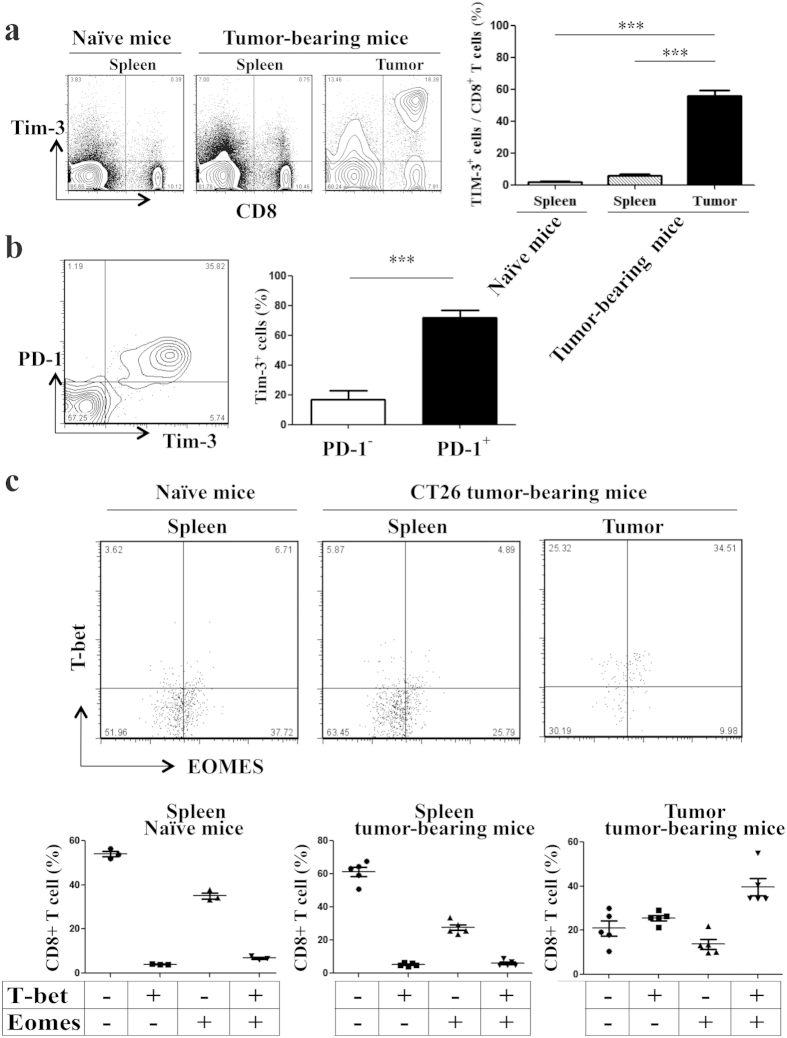
TIM-3 and PD-1 expression with T-bet and Eomes co-induction of tumor infiltrating CD8+ T cells in mouse CT26 colon tumor model. CT-26 tumor cells were inoculated subcutaneously in BALB/c mice and were allowed to form solid tumor. Mice were sacrificed on day 28- post tumor inoculation. Splenocytes from naïve non tumor-bearing mice served as controls. (**a**) Majority of tumor-infiltrating CD8+ T cells express TIM-3. CD3+ cells were analyzed for CD8 and TIM-3 expression. (**b**) Majority of tumor-infiltrating TIM-3+ CD8+ T cells co-expresses PD-1. (**c**) Majority of tumor-infiltrating CD8+ T cells are with co-induced of T-bet and Eomes. Data are mean ± SD of at least 3 similar experiments (n = 6; *** = p < 0.0001; p values for Mann-Whitney U test).

Similar to the human colon cancer tissues, TIM-3 was highly expressed on tumor-infiltrating CD8+ T cells in our mouse CT26 colon tumor model^25^,^26^,^27^. On day 28- post tumor inoculation, about 60% tumor-infiltrating CD8+ T cells expressed TIM-3 on their surface (Fig. 2a). Majority of the TIM-3+ tumor-infiltrating CD8+ T cells (>75%) also expressed PD-1 (Programmed cell death 1) on their surface (Fig. 2b). As exhausted CD8+ T cells are known to co-express TIM-3 and PD-1 (refs 7, 8, 9, 10, 11, 12) and co-induce T-bet and Eomes with terminal differentiation^28^, we studied T-bet and Eomes levels in our tumor-infiltrating CD8+ T cells. The frequency of T-bet-Eomes co-induced TIM-3 expressing cells was higher in tumor-infiltrating relative to splenic CD8+ T cells in the tumor-bearing mice (Fig. 2c). TIM-3 expression and T-bet-Eomes co-induction were minimum in the splenic CD8+ T cells in naïve mice, serving as control (Fig. 2c). Taken together, tumor-infiltrating CD8+ T cells are functionally competent despite co-expression of TIM-3 and PD-1, and co-induction of T-bet and Eomes in mouse CT26 colon tumor model.

### More effector cytokine secretion by TIM-3+ compared to TIM-3- population of tumor infiltrating CD8+ T cells in mouse CT26 colon tumor model

**Figure 3:**
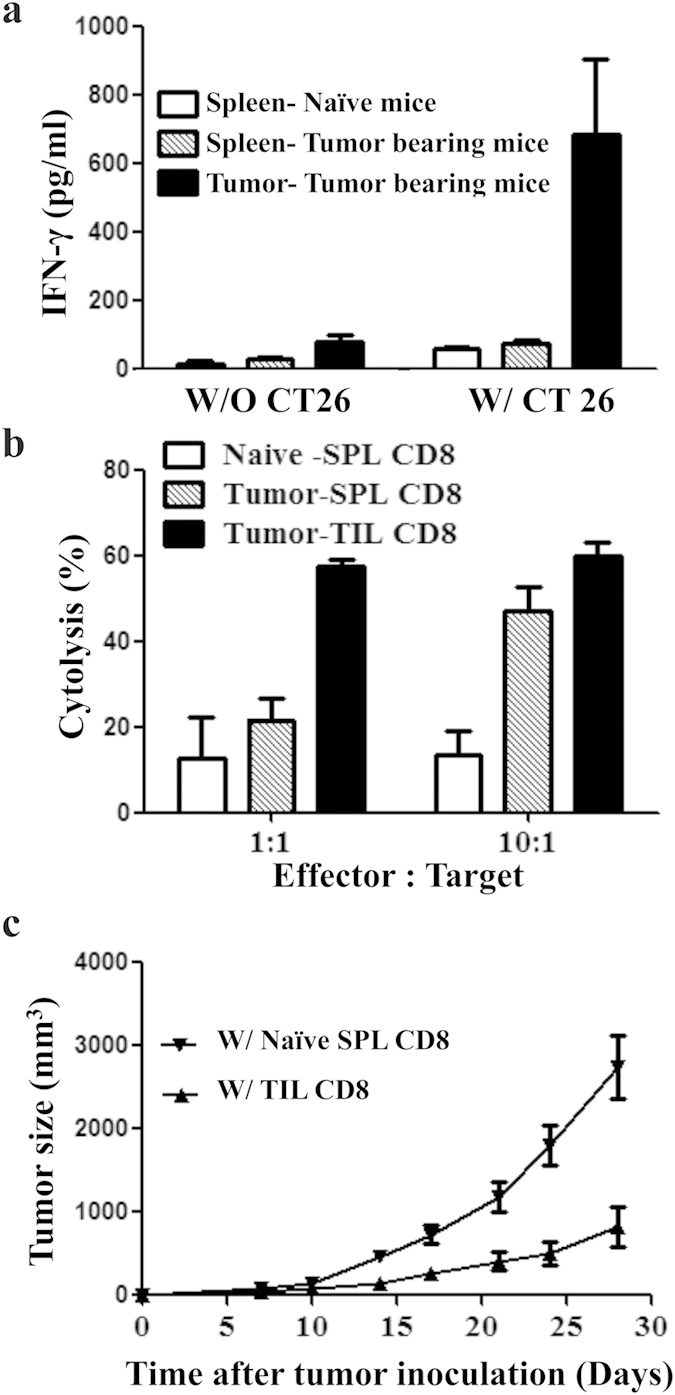
Tumor infiltrating CD8+ T cells are functionally active and inhibit tumor growth in mouse CT26 colon tumor model. CT-26 tumor cells were inoculated in BALB/c mice and tumor-infiltrating CD8+ T cells were sorted on day 28- post tumor inoculation. (**a**) Tumor-infiltrating CD8+ T cells secrete IFN-γ following culture with irradiated CT-26 tumor cells. Splenic CD8+ T cells from the tumor-bearing mice secrete minimum IFN-γ. (**b**) Both tumor-infiltrating and splenic CD8+ T cells of tumor-bearing mice execute cytolysis activity on CT26 tumor cells *in vitro*. The effect is higher by tumor-infiltrating compared to splenic CD8+ T cells. Splenic CD8+ T cells from naïve non tumor-bearing mice execute minimum cytolysis, served as control. (**c**) Upon adoptive transfer in another tumor-bearing mice, the tumor-infiltrating CD8+ T cells inhibit tumor growth. Transfer of naïve CD8+ T cells from spleens of non tumor-bearing naïve mice served as control. Data are mean ± SD of at least 3 similar experiments.

**Figure 4:**
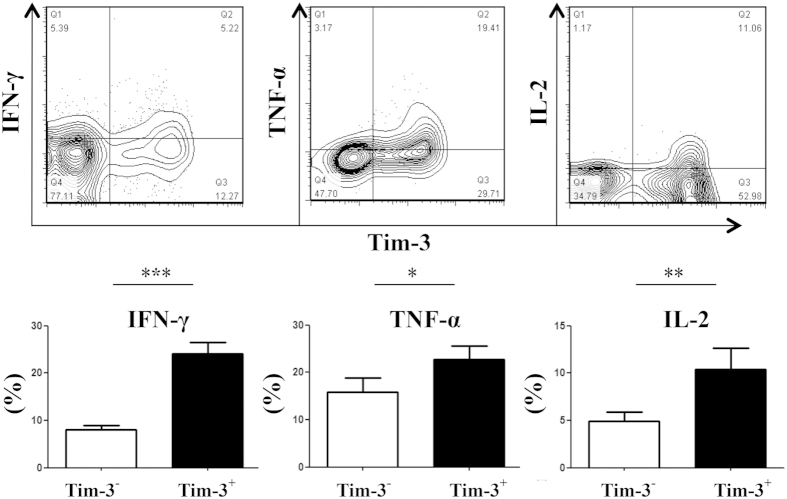
More effector cytokine secretion by TIM-3+ compared to TIM-3- population of tumor infiltrating CD8+ T cells in mouse CT26 colon tumor model. CT26 tumor cells were inoculated in BALB/c mice and were allowed to form solid tumor. The TIM-3+ population of tumor-infiltrating CD8+ T cells produces higher level of IFN-γ, TNF-α and IL-2 compared to that by the TIM-3- population on day 28- post tumor inoculation. Dot-plots demonstrate cytokine frequencies of TIM-3+ and TIM-3- populations of a single mouse representing each experimental group. Data are mean ± SD of at least 3 similar experiments (n = 6; *** = p < 0.0001; ** = p < 0.001; * = p < 0.01; p values for Mann-Whitney U test).

Majority of the tumor-infiltrating CD8+ T cells were functionally efficient despite surface co-expression of TIM-3 and PD-1 with co-induction of T-bet and Eomes (Fig. 2). The sorted tumor-infiltrating CD8+ T cells, from tumor-bearing mice on day 28- post tumor inoculation, secreted IFN-γ upon stimulation with irradiated CT26 tumor cells (Fig. 3a), executed cytolysis effect on CT 26 tumor cells (Fig. 3b) *in vitro*, and inhibited tumor growth after being adoptively transferred into mice receiving subcutaneous tumor inoculation on previous day (Fig. 3c). Splenic CD8+ T cells of tumor-bearing mice secreted basal level IFN-γ, executed minimum *in vitro* cytotoxic activity and *in vivo* tumor inhibition, and served as experimental control (Fig. 3a–c). Among the tumor-infiltrating CD8+ T cells, TIM-3+ cells produced more IFN-γ, TNF-α and IL-2 compared to that by TIM-3- cells when tested by intracellular staining on day 28- post tumor inoculation (Fig. 4). These results imply that TIM-3+ population is functionally as good as TIM-3- population of tumor-infiltrating CD8+ T cells.

### Higher percentage of apoptotic cells in TIM-3+ relative to TIM-3- population of tumor-infiltrating CD8+ T cells in CT26 mouse colon tumor model

**Figure 5:**
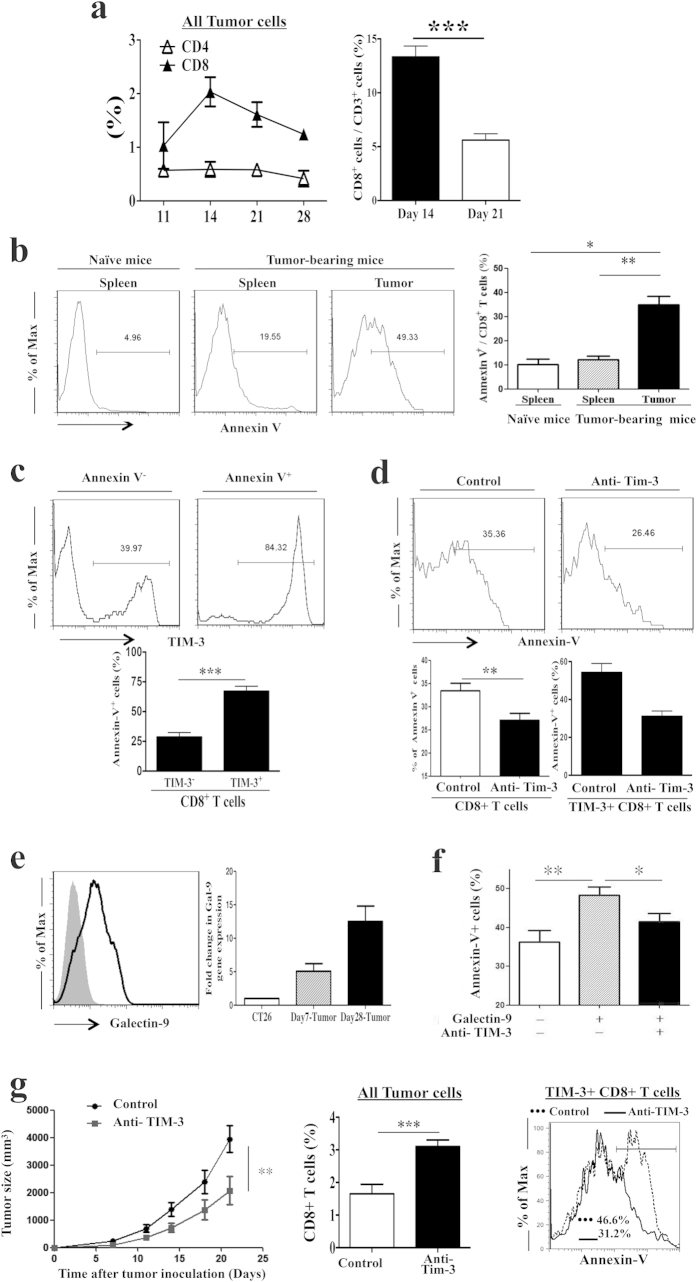
Tumor-derived galectin-9 acts on TIM-3 and induces apoptosis of TIM-3+ tumor-infiltrating CD8+ T cells in CT26 mouse colon tumor model. CT-26 tumor cells were inoculated in BALB/c mice and were allowed to form solid tumor. (**a**) The frequency of tumor-infiltrating CD8+ T cells decreases with tumor progression, as revealed by the comparison of CD8+ or CD4+ T cell percentages in all cells of the single-cell suspension of tumors on the stated days post tumor inoculation. (**b**) Higher apoptosis of tumor-infiltrating compared to splenic CD8+ T cells of tumor-bearing mice on day 28- post tumor inoculation. Splenic CD8+ T cells from naïve mice served as control. (**c**) Higher TIM-3 expression in apoptotic compared to non-apoptotic population of tumor-infiltrating CD8+ T cells on day 28- post tumor inoculation. (**d**) Decreased apoptosis of tumor-infiltrating CD8+ T cells, including the TIM-3+ CD8+ T cells, following *in vitro* culture with Anti- TIM-3 antibody. (**e**) CT26 tumor cells secrete galectin-9, as measured in tumor tissues on the stated days post tumor inoculation. (**f**) Recombinant galectin-9 increases and anti- TIM-3 antibody decreases apoptosis of tumor-infiltrating CD8+ T cells following *in vitro* culture. (**g**) Anti- TIM-3 antibody treatment inhibits tumor growth in mice. The treatment increases tumor-infiltrating CD8+ T cell frequency, which is associated with decreased apoptosis of TIM-3+ CD8+ T cells on day 21- post tumor inoculation. Data are mean ± SD of at least 3 similar experiments (n = 6; *** = p < 0.0001; ** = p < 0.001; * = p < 0.01; p values for Mann-Whitney U test).

Kinetic study revealed a decreased proportion of infiltrating CD8+ T cells relative to the total cells of a tumor during the progression with time (Fig. 5a). Such decrease could be a result of either decreased infiltration or increased death of CD8+ T cells in the tumor microenvironment. We analyzed death of tumor-infiltrating CD8+ T cells through apoptosis by the definition of Annexin-V binding to the exposed phosphatidylserine on the cell surface. Finding with annexin-V binding was validated with the measurement of caspase-3 activation in the cytosol. dUTP labeling (TUNEL assay) was used as a measure of DNA fragmentation. Compared to the splenic naive CD8+ T cells, the proportion of annexin-V positive cells were higher on tumor-infiltrating CD8+ T cells but not on the splenic CD8+ T cells of tumor-bearing mice on day 28- post inoculation (Fig. 5b). More importantly, annexin-V+ cells were more in the TIM-3+ compared to the TIM-3- population of tumor-infiltrating CD8+ T cells (Fig. 5c, Supplementary Fig. 1). Activation of caspase-3 and fragmentation of DNA were higher in the TIM-3+ compared to TIM-3- population of tumor-infiltrating CD8+ T cells as well (Fig. 5c, Supplementary Fig. 1). *In vitro* culture with anti- TIM-3 antibody reduced the Annexin-V binding to the surface of tumor-infiltrating CD8+ T cells implying reduced apoptosis of these cells. The reduction of apoptosis was associated with the less apoptosis of TIM-3+ population of the CD8+ T cells (Fig. 5d). These results suggest that TIM-3 plays an important role in the apoptosis of tumor-infiltrating CD8+ T cells.

### Tumor-derived galectin-9 induces apoptosis of tumor-infiltrating TIM-3+ CD8+ T cells in CT26 mouse colon tumor model

The CT26 tumor cells synthesized galectin-9, which was secreted into the tumor tissue of tumor-bearing mice (Fig. 5e). Addition of recombinant galectin-9 increased apoptosis of the tumor-infiltrating CD8+ T cells when the cells were sorted from tumor-bearing mice on day 28- post tumor inoculation and cultured *in vitro*. The galectin-9 induced apoptosis was reduced significantly with added anti-TIM-3 antibody in the culture (Fig. 5f). As TIM-3 blockade attenuated galectin-9 induced apoptosis of tumor-infiltrating CD8+ T cells *in vitro*, we then tested whether this attenuation of apoptosis could be translated into improved tumor inhibition *in vivo*. CT26 tumor-bearing mice were treated with anti-TIM-3 or control antibody. Treatment of anti-TIM-3 antibody reduced tumor size, which was associated with increased tumor-infiltrating CD8+ T cells owing to decreased apoptosis of TIM-3+ CD8+ T cells (Fig. 5g). Taken together, tumor-derived galectin-9 induces apoptosis in tumor-infiltrating CD8+ T cells and blockade of TIM-3 attenuates the apoptosis and augments anti-tumor activity of infiltrating CD8+ T cells.

### TIM-3 blockade enhances therapeutic efficacy of cyclophosphamide

**Figure 6:**
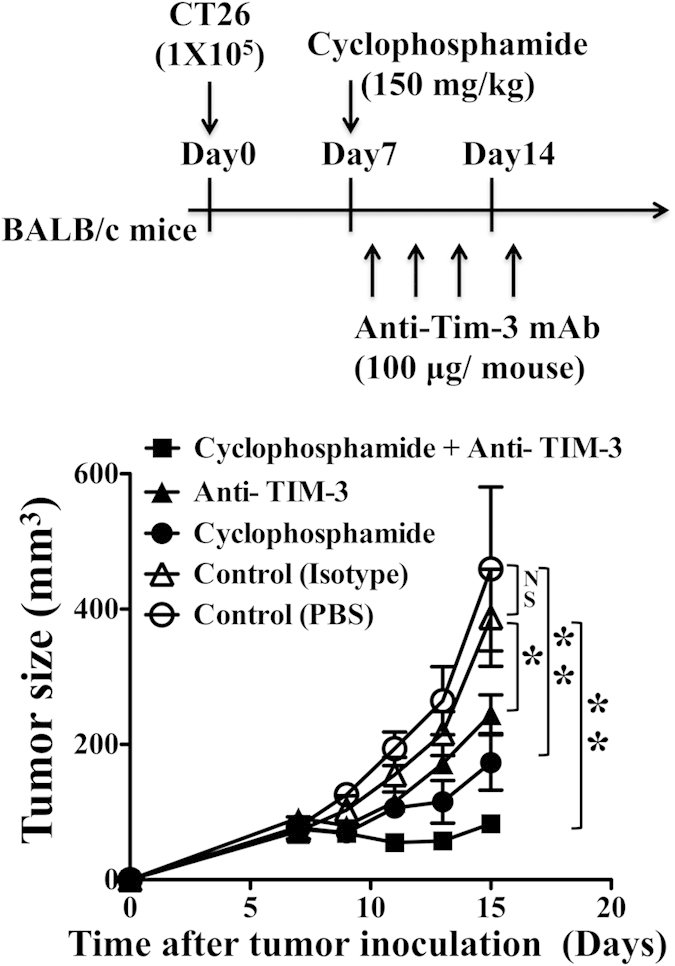
TIM-3 blockade enhances therapeutic efficacy of cyclophosphamide to inhibit tumor growth in mice. CT-26 tumor cells were inoculated in mice, allowed to form solid tumor and treated with stated molecules as mentioned in the figure. Treatment of anti-TIM-3 antibody or cyclophosphamide alone inhibits tumor growth. Combined therapy with anti-TIM-3 antibody and cyclophosphamide causes greater inhibition of tumor growth. Tumor-bearing mice with treatment of isotype antibody or PBS served as controls (n = 6; mean ± SEM; ** = p<0.001; * = p < 0.01; NS = Non significant, p > 0.05; p values for Mann-Whitney U test).

The treatment of cyclophosphamide, a common chemotherapeutic agent^29^,^30^, is often been combined with other immunotherapy to achieve better outcome. We found that TIM-3 blockade augmented therapeutic efficacy of cyclophosphamide in CT26 mouse colon tumor model. Both the treatments either with cyclophosphamide or with anti-TIM-3 antibody inhibited tumor growth in mice when CT26 tumor cells were allowed to form the tumor mass for initial 7 days before starting the treatment. The inhibition of tumor growth was much more significant with combined therapy of cyclophosphamide treatment from day-7 and anti- TIM-3 treatment from day-8 post tumor inoculation (Fig. 6). Treatment with PBS or isotype antibody, as controls for both cyclophosphamide and anti-TIM-3 antibody, did not show any effect on tumor growth. These results imply that TIM-3 blockade augments anti-tumor effect of cyclophosphamide *in vivo*.

## Discussion

Our data reveals cancer tissue resident CD8+ T cells are functionally competent for effector cytokine, IFN-γ, response although majority of them express TIM-3 on their surface in humans suffering from colorectal cancer. The cancer tissue resident TIM-3+ CD8+ T cells are prone to death and perhaps the elimination of functionally efficient CD8+ T cells contributes for immune dysfunction in the cancer tissues in humans. We have seen similar phenomenon of functional efficiency including effector cytokine secretion, cytolysis activity and *in vivo* tumor inhibiting capacity of tumor-infiltrating CD8+ T cells in CT26 mouse model of colon cancer. Majority of the tumor-infiltrating CD8+ T cells express TIM-3 and the functional efficacy is greater in the TIM-3+ relative to the TIM-3- population. The tumor cells secrete galectin-9 in the tumor microenvironment that act on TIM-3 to induce apoptosis in the tumor-infiltrating CD8+ T cells. TIM-3 blockade reduces galectin-9 driven apoptosis in tumor-infiltrating TIM-3+ CD8+ T cells and inhibits tumor growth in mice. The blockade enhances therapeutic efficacy of cyclophosphamide to treat tumor in mice as well.

Expression level of TIM-3 has been shown to correlate with the exhaustion level of CD8^+^ T cells in chronic viral infections^31^,^32^ and in consequence, TIM-3 expression claims as the marker for exhausted CD8^+^ T cells in tumors of humans and mice^9^,^10^,^33^. Perhaps the co-induction of transcription factors T-bet and Eomes symbolizes terminal differentiation in the TIM-3+ CD8+ exhausted T cells^28^. The CD8+ T cells may still holds some tumor inhibition ability in the tumor microenvironment as their increase in number predicts a better outcome in humans suffering from colon cancer^1^,^34^. The so-called exhausted CD8+ T cells can constrain viral replication too^35^. Our results provide evidences for preserved effector function of tumor-infiltrating CD8+ T cells as well. Although T-bet and Eomes are co-induced in majority, our findings of better effector functions of TIM-3+ relative to TIM-3- population of CD8+ T cells are similar to the previous findings in infectious diseases^14^,^18^. However, the effector function of tumor-infiltrating CD8+ T cells is not sufficient to inhibit the growth of that tumor. Tumor inhibition can only be achieved with transfer of cells into other tumor-bearing mice, suggesting an increase in tumor-infiltrating CD8+ T cell frequency enhances effector function and tumor inhibition.

Our results reveal a decrease of CD8+ T cells in tumor microenvironment reduces tumor inhibitory effector function and thereby helps in tumor progression. This is perhaps due to apoptosis of tumor-infiltrating CD8+ T cells, especially TIM-3+ CD8+ T cells, similar to that in human cancers including ‘bladder’ and ‘head and neck’ cancers^36^,^37^. TIM-3 is known to participate in the apoptosis of CD8+ T cells in chronic virus infections too^38^. The galectin-9 is arguably a ligand of TIM-3 (refs 16, 39, 40, 41), which increases apoptosis of effector T cells in autoimmune diseases and in both chronic and acute viral infections^16^,^18^,^38^,^42^,^43^. The CT26 tumor cells secrete galectin-9 which causes apoptosis of tumor-infiltrating CD8+ T cells in the tumor microenvironment in our experiment. Blockade of TIM-3 by anti- TIM-3 antibody reduces apoptosis of tumor-infiltrating TIM-3+ CD8+ T cells and inhibits tumor growth in mice. The blockade also reduces the CD8+ T cell apoptosis caused by exogenous galectin-9. The blockade of TIM-3/galectin-9 interaction enhances the therapeutic potential of cyclophosphamide to inhibit tumor growth in mice as well. Our results imply that tumor-infiltrating TIM-3+ CD8+ T cells still hold competent effector functions. However, apoptosis of TIM-3+ CD8^+^ T cells through their TIM-3 and tumor-derived galectin-9 reduces effector cell frequency and thereby helps tumors to escape from the immune attack.

Cyclophosphamide is often used for the treatment of cancer as it inhibits regulatory T cells, enhances T cell proliferation and survival, promotes Th17 differentiation and cytokine production and in addition, resets dendritic cell homeostasis^29^,^44^,^45^. Immunotherapy with cyclophosphamide is common in animal models and in clinical trials since 1988 (refs 46, 47). Combined treatment of OX40 receptor ligands or PD-1 antagonists with cyclophosphamide favors the outcome^48^,^49^. Here we reveal better outcome with anti- TIM-3 antibody therapy in combination with the cyclophosphamide treatment in mice. This needs to be explored further for novel scheme design of cancer immunotherapy in humans.

## Methods

### Study population

A total of 7 human colon cancer tissues and peripheral blood samples were collected immediately after a regular colon cancer operation for patients with colorectal cancer (CRC). About 1 × 1 cm^2^ size tumor specimens from each patient were used for the present study. All patients in this study had provided written informed consent. This study protocol was conformed to the ethical guidelines of the Declaration of Helsinki, 1975; and was approved by the ethical committees of Chang Gung Memorial Hospital.

### Mice

BALB/c mice were purchased from the National Laboratory Animal Center of Taiwan. All mice were maintained in the animal house of Chang Gung Memorial Hospital and used in experiment at age 8–10 weeks. All animal breeding and experiments were in accordance with guidelines of the institutional animal ethics committee. All experimental protocols were approved by ‘The Animal Care and Use Committee’ of the ‘Chang Gung Memorial Hospital’, Taiwan. The committee recognizes that all the experiments involving the use of mice were carried out in strict accordance with the Animal Protection Law by the Council of Agriculture, Executive Yuan, Taiwan, R.O.C., and the guideline as shown in the guide for the Care and Use of Laboratory Animals as promulgated by the Institute of Laboratory Animal Resources, National Research Council. USA.

### Cell lines and tumor model establishment

Mouse CT26 colon tumor model^25^,^26^,^27^ was used in this study. The murine colon carcinoma cell line, CT26, was purchased from the American Type Culture Collection (Union Biomed Inc., Taipei, Taiwan) and was maintained in our laboratory. Cells were regularly cultured in RPMI1640 medium containing 10% FBS. CT26 tumor cells were inoculated subcutaneously (1 × 10^5^) to BALB/c mice. Tumor diameters were measured twice a week. Tumor size was calculated according to the formula: V = [(length × width^2^)/2].

### Antibodies and flow cytometry

All antibodies were purchased from BD Biosciences, eBiosciences or Bio-legend. Single cell suspensions were stained for surface or intracellular proteins and cytokines according to the manufacturer’s instructions. For intracellular cytokine staining, 1 × 10^6^ cells per well were stimulated with 50 ng ml^−1^ phorbol 12-myristate 13-acetate (PMA) and 500ng ml^−1^ ionomycin in the presence of Golgistop (BD Biosciences) for 5 hours, followed by surface and intracellular staining. With surface staining of CD3, CD8 and TIM-3, the measurements of Annexin-V binding (FITC Annexin-V apoptosis detection kit, Cat# 556547, BD Pharmingen), caspase-3 activation (FITC active caspase-3 apoptosis kit, Cat# 550480, BD Pharmingen) and DNA fragmentation (APO-DIRECT kit, Cat# 556381, BD Pharmingen) were done according to the manufacturer’s instruction. Stained cells were acquired through FACSCalibur (BD Biosciences) and analyzed using CellQuest Pro or FlowJo software.

### Cell isolation

Human peripheral blood mononuclear cells were separated from blood samples by Ficoll density gradient (Pharmacia). Tumors were chopped into small pieces using a razor blade and incubated with collagenase type IV (0.1%; Sigma-Aldrich) in Hank’s Balanced Salt Solution (HBSS) for 30 min at 37 °C. After passing through nylon mesh, single-cell suspension was separated with Ficoll, and leukocytes were recovered from the interphase.

### CD8+ T cell purification

Single cell suspensions were prepared from spleens or tumors of naive mice or day-28 CT26 tumor-bearing mice. CD8+ T cells were isolated using magnetic micro beads conjugated with anti-mouse CD8 by AutoMACS (Miltenyi Biotec) according to the manufacturer’s instructions. Cell purity (>90%) for all populations was confirmed by flow cytometry.

### ELISA for IFN-γ secretion by tumor infiltrating CD8+ T cells

Purified CD8+ T cells (1 × 10^5^ cells) and mitomycin C-treated syngeneic BALB/c T cell-depleted splenocytes (1 × 10^5^ cells) were mixed with or without irradiated CT26 tumor cells (1 × 10^3^ cells) and incubated in 96-well culture plates for 48 hours at 37 °C. Supernatant were collected and assayed for IFN-γ production by ELISA according to manufacturer instructions (BD Bioscience).

### Cytolysis activity assay

Target cells (CT26 tumor cells) were radiolabeled with [methyl-^3^H] thymidine (Perkin Elmer) to a concentration of 5 μ Ci ml^−1^ for 24 hours at 37 °C before cytotoxicity assay. A serial dilution of effector cells (CD8+ T cells) was mixed with labeled target cells in 96-well culture plates for overnight incubation. The culture plate was harvested for beta radiation counting. % cytotoxicity = [(S − E)/S] × 100 were calculated. E: number of cells in experimental wells; S: number of cells in spontaneous release wells.

### Adoptive transfer of CD8+ T cells

Purified CD8+ T cells from day-28 CT26 tumor-bearing mice or naïve mice in appropriate numbers (3 × 10^5^) were re-suspended in 0.2 ml of HBSS and then injected into mice with one day tumor-bearing through the tail vein.

### Quantitative RT-PCR

Cell lines or tumor tissues were homogenized with 1 ml TRI Reagent to extract total RNA. The concentration of RNA was determined spectrophotometrically and quantitative RT-PCR was performed as previously described^50^.

### Galectin-9-induced apoptosis assay

CD8+ T cells, havested from TILs of day-21 CT26 tumor-bearing mice, were incubated in media alone or stimulated with 5 ug ml^−1^ recombinant galectin-9 (ProSpec) in the absence or the presence of 10 ug ml^−1^ anti-TIM-3 mAb (clone RMT 3–23; Bio-X-Cell) for 5 hr at 37 °C. Cells were then stained for annexin V using FITC Annexin V Apoptosis Detection Kit (BD Bioscience). Stained cells were analyzed immediately by flow cytometry.

### TIM-3 blockade in tumor-bearing mice

For TIM-3 blockade experiments, CT26 tumor-bearing mice were treated with intraperitoneal injection of anti-TIM-3 mAb (100 μg/mouse; Catalog#: BE0115, Clone: RMT3–23, Bio X Cell, NH 03784-1671 USA) on day 7, 9, 11 after tumor inoculation. Expression levels of annexin V and IFN-γ in tumor-infiltrating CD8+ T cells were determined by flow cytometry on the days mentioned in the text. For combined therapy, tumor-bearing mice were treated with cyclophosphamide (Sigma) on day 7 and anti-TIM-3 mAb on day 8, 11, 13, and 15 after tumor inoculation. Tumor-bearing mice receiving treatment of cyclophosphamide or anti-TIM-3 mAb alone served as controls. Tumor-bearing mice with treatment of rat IgG2a isotype antibody (Bio X cell, Catalog#: BE0089, Clone: 2A3) or PBS served as experimental controls.

### Statistical analysis

Mann-Whitney U test was used for statistical analyses of difference between groups, and a paired t test was used to determine pairwise differences. All calculations were made using PRISM (version 5.00; GraphPad Software) and a value *p* < 0.05 was considered as significantly different.

## Additional Information

**How to cite this article**: Kang, C.-W. *et al*. Apoptosis of tumor infiltrating effector TIM-3+CD8+ T cells in colon cancer. *Sci. Rep*. **5**, 15659; doi: 10.1038/srep15659 (2015).

## Supplementary Material

Supplementary Information
