# Supplementary material for: Apoptosis of tumor infiltrating effector TIM-3+CD8+ T cells in colon cancer
Source: Sci Rep. 2015 Oct 23;5:15659. doi: 10.1038/srep15659 (PMC4616166; doi:10.1038/srep15659)
Supplement: Supplementary Information [file srep15659-s1.pdf]

Apoptosis of tumor infiltrating effector TIM-3+CD8+ T cells in colon cancer

Chiao-Wen Kang, Avijit Dutta, Li-Yuan Chang, Jayashri Mahalingam, Yung-Chang Lin, Jy-Ming Chiang, Chen-Yu Hsu, Ching-Tai Huang, Wan-Ting Su, Yu-Yi Chu, Chun-Yen Lin.

Supplemental Figures

Supplementary Figure 1

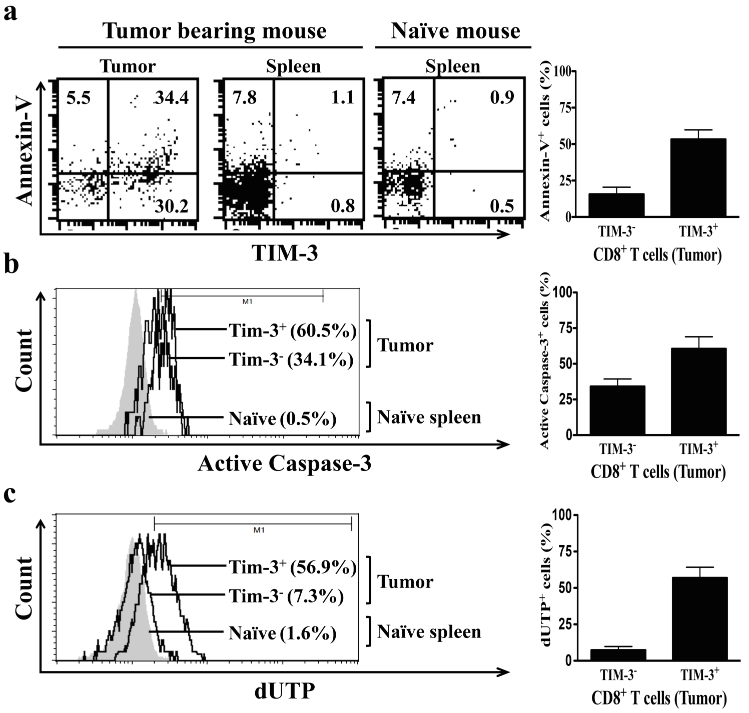

Increased apoptosis of TIM-3+ compared to TIM-3- tumor-infiltrating CD8+ T cells in CT26 mouse colon tumor model. CT-26 tumor cells were inoculated in BALB/c mice and were allowed to form solid tumor. Higher percentage of (A) Annexin-V (B) active Caspase-3 and (C) d-UTP positive cells in TIM-3+ compared to TIM-3- population of tumor-infiltrating CD8+ T cells on day 14- post tumor inoculation. Splenic CD8+ T cells from naïve mice served as control. Data are mean ± SD.
